# How do community-based eye care practitioners approach depression in patients with low vision? A mixed methods study

**DOI:** 10.1186/s12888-019-2387-x

**Published:** 2019-12-30

**Authors:** Claire Nollett, Rebecca Bartlett, Ryan Man, Timothy Pickles, Barbara Ryan, Jennifer H. Acton

**Affiliations:** 1 Centre for Trials Research, Cardiff University, 4th Floor, Neuadd Meirionnydd, Heath Park, Cardiff, CF14 4YS UK; 2 School of Optometry and Vision Sciences, College of Biomedical and Life Sciences, Cardiff University, Maindy Road, Cardiff, CF24 4HQ UK; 3 Singapore Eye Research Institute, 20 College Road, The Academia, Discovery Tower Level 6, Singapore, 169856 Singapore

**Keywords:** Vision impairment, Low vision, Depression, Screening, Practitioners, Training, Confidence, Barriers

## Abstract

**Background:**

Clinically significant depressive symptoms are prevalent in people attending low vision clinics and often go undetected. The Low Vision Service Wales (LVSW) plans to introduce depression screening and management pathways. Prior to implementation there is an unmet need to understand how eye care practitioners providing the service currently address depression with patients, and the characteristics and beliefs that influence their practice.

**Methods:**

A mixed methods convergent design was employed. Twelve low vision practitioners were purposively selected to engage in individual semi-structured interviews which were analysed using thematic analysis. A further 167 practitioners were invited to complete a questionnaire assessing professional background, current practice, confidence and perceived barriers in working with people with low vision and suspected depression. Multiple regression analyses were performed to determine the characteristics related to the Rasch-transformed questionnaire scores.

**Results:**

Of the 122 practitioners that responded to the questionnaire, 33% aimed to identify depression in patients, and those who were more confident were more likely to do so. Those who scored higher on the perceived barriers scale and lower on confidence were less likely to report acting in response to suspected depression (all *p* < 0.05). Three qualitative themes were identified; depression is an understandable response to low vision, patients themselves are a barrier to addressing depression and practitioners lacked confidence in their knowledge and skills to address depression. The qualitative data largely expanded the quantitative findings.

**Conclusions:**

Practitioners viewed their own lack of knowledge and confidence as a barrier to the identification and management of depression and expressed a need for training prior to the implementation of service changes. The study findings will help to inform the development of a training programme to support low vision practitioners and those working with other chronic illness in Wales, and internationally, in the identification and management of people with depression.

## Background

Depression is common in the general population, but is more prevalent in people with chronic illnesses such as hypertension, diabetes and stroke: they are at least twice as likely to develop depression [1, 2]. For those with multi-morbidity, defined as two or more chronic conditions, the risk is three time as great [3]. People with low vision are also a high risk group. In the UK, over 2 million people are living with sight loss [4], with 77% of people affected aged 65 or over [5], and co-morbidity with other chronic health conditions is common [6]. A significant subset of people with sight loss are categorised as having “low vision”, which can be defined as having an impairment in vision that cannot be fully corrected with glasses, contact lenses or medical intervention and causes restriction in a person’s everyday life [7]. The leading causes of low vision globally are eye diseases including age-related macular degeneration and glaucoma [8]. In 2015, an estimated 129 million people globally were living with low vision [8] and in the UK, around 1.3 million people are currently affected [4].

There is a well-established link between low vision and depression: people with low vision are 2–5 times more likely to experience depression or significant depressive symptoms [9–11]. For example, a large population based study of older adults in the UK found that the prevalence of significant depressive symptoms in those with low vision was 13.5% (compared to 4.6% in those with good vision) [9]. In those attending low vision rehabilitation clinics, 37–43% were found to have significant depressive symptoms [12, 13], and the prevalence of Major Depressive Disorder was 5.4% (compared to 1.2% in people with normal sight) [10]. One explanation for the increased risk in this group is the Activity Restriction Model of Depressed Affect [14], which posits that depression results from having to relinquish valued activities. Vision loss is known to lead to high levels of functional impairment, impacting on activities of daily living [11] and engagement in hobbies and social activities [15, 16]. This impairment is likely compounded by co-morbidity with other chronic conditions such as diabetes and stroke, both of which are more prevalent in people with low vision [6].

The presence of depression in people with chronic conditions can lead to poorer treatment adherence [17] and engagement in rehabilitation, resulting in poorer overall outcomes [18, 19] and increased functional disability and health resource utilisation [2]. It is important depression is diagnosed and treated, however, depression often goes undetected by clinicians [20]. Some people with depression, particularly older adults, fail to present with low mood and instead report non-specific or somatic symptoms such as change in appetite, sleep problems or low energy [21]. In elderly patients or those with chronic conditions, it is easy for clinicians to mistakenly attribute these symptoms to the physical illness or ‘old age’, thereby missing depression [18, 22]. These views are often held by elderly patients themselves [23]. In addition, they have difficulties expressing their moods [24] and beliefs around stigma which may prevent them from seeking help [25], compounding the chances of under-recognition by primary care clinicians who may not possess the skills or confidence to detect depression [23]. Finally, older adults with poor vision are among those least likely to be recognised as having depression in primary care [26].

To address under-detection of depression, several U.S. and Canadian national guidelines recommend routine screening for depression in people with chronic illness [27–29]. The UK’s National Institute of Health and Care Excellence (NICE) advises practitioners working in primary care and in general hospital settings to be aware that patients with a chronic physical health problem are a high risk group, particularly where there is functional impairment, and that they should be alert to possible depression [19]. They suggest practitioners consider asking patients two screening questions (known as the Whooley questions) [30], with referral for assessment if the result is positive. There is much debate about the pros and cons of routine screening for depression. Evidence suggests it can lead to diagnosis of new cases and early intervention [31], however this will only occur when provided alongside effective management strategies [32]. Potential harms include identifying false positives, possibly leading to unnecessary distress and wasted resources [33], and an increase in consultation time [33]. Moreover, whilst screening using a short validated tool appears to be a simple procedure, it is in fact a more complex intervention when screening for depression [34, 35]. Alderson et al. [34] identified five barriers to screening for depression in chronic health settings presented by staff, patients and systems, and recommend that all those involved need to be prepared in advance of the introduction of screening into a service. With regard to professionals, they suggest examining their attitudes towards and skills in detecting depression prior to implementation.

The Low Vision Service Wales (LVSW) is a national community care-based rehabilitation service in Wales, UK, delivered in community optometry practices by 193 low vision practitioners. The prevalence of clinically significant symptoms in patients attending the service was found to be 39% [13] and 75% of those identified were not receiving treatment. Consequently, and in line with government guidance documents [19, 36], the LVSW plans to introduce depression screening and management pathways. As noted above, prior to implementation there is a need to understand the beliefs, skills [34] and current practice of community-based low vision practitioners around depression screening and management. Little is known about whether they are already addressing the subject of depression with patients, and if so, how.

A qualitative study conducted in a tertiary eye care hospital in Melbourne reported on eye care practitioners’ beliefs, practice and perceived barriers to working with depression [37] and a further series of quantitative studies with eye health professionals in hospitals and private practice in Australia [38–40] concluded that interventions, including training programs, are required to improve depression management within eye care services. Aside from these studies, there is a paucity of evidence in this area, particularly in regard to community and UK based low vision practitioners.

Therefore, there is a need to understand: if/how community low vision practitioners currently identify and manage depression and the characteristics, beliefs and barriers linked to their practice, prior to the introduction of routine screening in low vision services. In addressing these knowledge gaps, the results will help to inform the development of a training programme to support low vision practitioners in Wales, and internationally, to screen and manage people with low vision and depression. Our specific research questions were:

1. What is community low vision practitioners’ current practice around identifying and responding to depression in patients with low vision?
2. What characteristics and beliefs are linked to their current practice?

## Methods

### Study design and participants

The study was granted ethical approval from the School Research Ethics Audit Committee at the School of Optometry & Vision Sciences, Cardiff University: ref. 1457. All participants were given information sheets about the study prior to providing consent and all practices followed the guidelines of the Declaration of Helsinki [41]. The study was carried out within the The study was carried out within the LVSW. The LVSW helps people with low vision to maintain their independence through provision of advice and support, prescribing optical and non-optical low vision aids such as magnifiers, signposting and referral to other services including voluntary organisations, social care and healthcare professionals. The service is provided by low vision practitioners who are eye care professionals (optometrists, dispensing opticians and an ophthalmic practitioner). In addition to the core training required for registration with their respective professional bodies, all practitioners are required to complete the College of Optometrists Certificate in Low Vision (course details [42]) and undertake a process of re-accreditation on a 3 yearly basis.

**Fig. 1:**
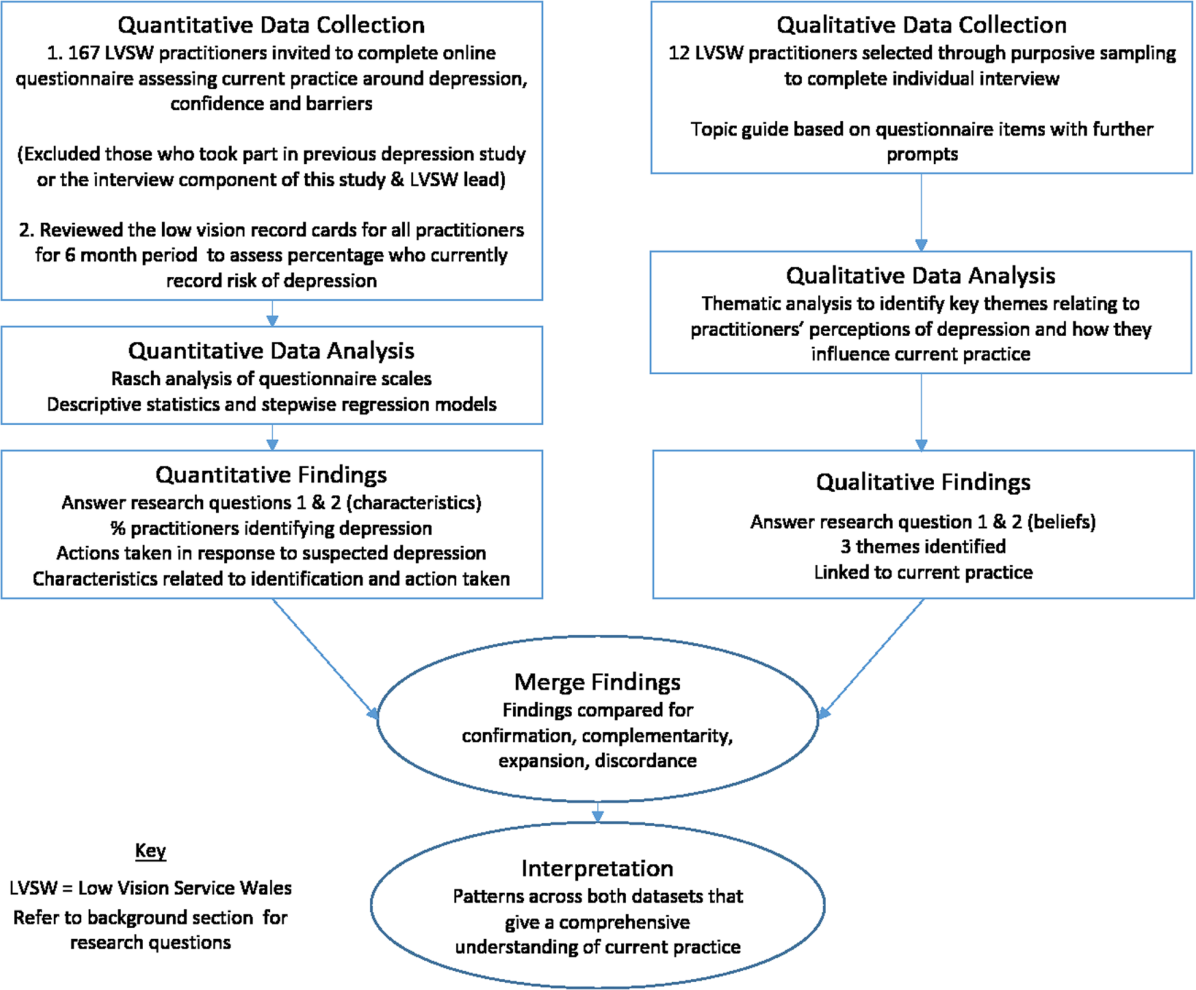
The Convergent Mixed Methods Design

This study employed a cross-sectional design using baseline data from an ongoing study. Given the paucity of previous relevant literature, we used a convergent mixed methods design [43] to obtain both a quantitative and qualitative understanding of current practice (see Fig. 1). The quantitative aspect included both a questionnaire and routinely collected data, to allow an investigation of general trends in clinical practice (behaviour) around addressing depression, whilst the qualitative individual interviews were used to explore in-depth personal perspectives on the subject (Research Question 1). The questionnaire was also used to examine associations between practitioner characteristics and practice, whilst the interviews sought to understand practitioner beliefs which influenced their practice (Research Question 2). The results from the two datasets were compared in a mixed methods analysis, thus providing a more comprehensive understanding than either method alone could give [43, 44].

Eligible participants included all practitioners accredited by LVSW, excluding 12 practitioners who previously received training in depression for a prior research study [45] and the Clinical Lead for the service (author RB) who is also trained in depression identification and management (*N* = 179). The practitioners were invited to take part in *either* the questionnaire (*N* = 167) or an interview (*N* = 12) to reduce the burden on practitioners, and to reduce the influence of bias from a prior response to the alternate method.

### Quantitative measures

#### Online questionnaire

We utilised four sections of a questionnaire developed for use with eye care practitioners and employed and validated in previous research [39] (See Additional file 1 – Study Questionnaire). The scales used in the questionnaire were developed from scales used with professionals working with the elderly. They were refined through focus groups with eye health professionals and validated using Rasch analysis (for a full description of original questionnaire development, refer to Rees et al. [39]). Part A of the questionnaire consisted of questions to record demographic information (age and gender) and professional/work-related characteristics. The latter included information on job role, place of work, length of registration/service, number of patients seen per month, time spent with patient and type of assessment (that is, do they provide practice based or domiciliary assessments, or a mixture of both). Part B of the questionnaire assessed the practitioner’s current practice in relation to working with patients with low vision and depression. Part B consisted of two items on the intention to identify depression in low vision patients and the use of a screening tool. This was followed by an 8-item “actions in practice” scale assessing actions taken in response to suspected depression (e.g. “Discuss their feelings with them”, “refer the patient to the GP”). Part C measured confidence in working with people with low vision and depression using an 11-item scale and Part D measured perceived barriers to working with patients with low vision and depression using a 13-item scale. Items were answered using Likert Scale response categories.

#### Low vision record card

Completion of a low vision record card by the LVSW practitioner is a requirement for every patient assessment conducted. It consists of clinical details of the patient and check boxes to indicate specific risks faced by the patient, including depression. There is currently no formal requirement for practitioners to screen for risk of depression. Hence, any instances of risk of depression being recorded are based on the practitioner’s own assessment: this may have occurred through use of a standardised screening tool if they are familiar with one, or it may be a more informal judgement.

### Qualitative interviews

In-depth semi-structured interviews were conducted with individual participants using a topic guide developed by the research team. The guide was designed to elicit information to answer the two research questions and to allow comparison with the data gained from the questionnaires. Four open-ended questions were based around the three questionnaire scales and asked about participants’ current practice around identifying and responding to depression in people with low vision, their confidence in working with people with depression and their perceived barriers. Four further questions examined their understanding and personal experience of depression, perceptions of their role and training needs. The guide was reviewed by the Qualitative Research Group (Centre for Trials Research, Cardiff University) and piloted with an optometrist not taking part in the study. As a result of both, some questions were re-worded to elicit specific examples and prompts were added to the main questions to encourage more detailed information in the instance the respondent was not forthcoming.

### Procedures

The aim of the qualitative interviews was to identify common patterns of beliefs and practice across LVSW practitioners. Given the variety in their demographic and professional characteristics, and that these characteristics may well influence their beliefs and practice, we selected potential interview participants using maximum variation sampling. This is a sampling strategy which aims to identify shared patterns across variations in participants [46] and involves selecting participants across a spectrum [47], in this case, of demographic and professional characteristics. The LVSW Clinical Lead reviewed the list of practitioners and selected a potential sample of participants based on a mix of demographic (eg. age, gender, location) and work-related (eg. length of service, job role) characteristics. Practitioners were emailed an invitation and Participant Information Sheet. To minimise the pressure to consent, interested practitioners were asked to contact an independent researcher (CN) and consenting participants remained anonymous to the Clinical Lead and other study team members. Twelve practitioners agreed to take part. Nine interviews were conducted on the telephone and three were undertaken face-to-face at the practitioner’s place of work or at the School of Optometry and Vision Sciences, Cardiff University. The participants provided written or verbal consent to take part and the interviews were audio-recorded. Most interviews lasted 30–40 min. All of the interviews were conducted by one author (CN), an experienced researcher who has a background in mental health research and practice, is independent from the LVSW and was unknown to the practitioners. Field notes were completed immediately after each interview and recorded: key impressions, emotions expressed by interviewee, reflections on the interview process, practical observations and beliefs or experiences of the interviewer which may have been relevant to the process. A reflexive journal was kept throughout the interview and analysis process.

The questionnaire was transferred into an online format hosted by Online Surveys [48]. It was tested and refined to maximise usability and quality of data collection. All practitioners were sent an email containing a link to the questionnaire and asked to complete it as part of a reflection task examining their current practice around depression. Reflection tasks are a standard part of the ongoing LVSW re-accreditation process and depression was a theme for 2018. In addition, the email contained a copy of the Participant Information Sheet, and practitioners were informed that if they were happy for their answers to also be used for research purposes, they could indicate their consent at the start of the questionnaire. From an ethical point of view, and because of the sensitive nature of the topic, their responses were anonymous so the Clinical Lead could not trace who had consented, thus minimising the pressure to agree to the research aspect. All practitioners were given 2 weeks to complete the questionnaire and a generic email reminder was sent after 1 week.

Data from all record cards completed by all practitioners (other than those excluded from the study) during the 6-month period from 1st July to 31st December 2017 were collated to determine the number of practitioners who identified a risk of depression in any instance. This would give a somewhat more objective indication of how many practitioners are currently considering and recording depression in their current practice over self-report on the questionnaires/interviews alone.

#### Psychometric assessments of questionnaire scales

Rasch analysis was used to assess the psychometric properties of the three quantitative questionnaire scales in Part B, C and D, using the Andrich rating scale model [49] with Winsteps software (version 3.92.1, Chicago, Illinois, USA). Further details of the methodology used and the psychometric properties of the three questionnaires can be found in Additional file 2 – Rasch Analysis Methodology & Results.

#### Statistical analysis

The questionnaire data were analysed using intercooled STATA Version 13 (StataCorp LLC, TX, USA). Descriptive statistics were used to describe the background characteristics of the sample (Part A) and the practitioners’ reported current practice in terms of identification of depression (two questions in Part B). Categorical variables were summarised as numbers and percentages, continuous variables as medians with interquartile ranges.

Two stepwise multiple regression analyses were performed to determine the characteristics related to current practice. The first was a stepwise multivariable logistic regression to examine the relationship between intention to identify depression (Yes/No based on participant response to the first question in Part B) and the practitioners’ background characteristics (Part A), confidence (Part C) and barriers scores (Part D). The results are presented using odds ratios (OR) with 95% confidence intervals and *p*-values*.* The second was a stepwise multivariable linear regression to examine the relationship between the “action in practice” scale score (Part B) and the practitioners’ background characteristics, confidence and barriers scores. The results are presented using effect sizes with 95% confidence intervals and *p*-values. With regards to the record card data, descriptive statistics were used to describe the number and percentage of practitioners who had recorded at least one instance of a patient being at risk of depression to determine how many practitioners identify and record depression as part of their current practice.

#### Qualitative analysis

The audio-recordings were transcribed verbatim (including non-verbal behaviour) by a professional transcription company. All transcripts were checked for accuracy against the original recording by the interviewer. CN conducted Thematic Analysis using Braun & Clarke’s approach [50]. The data were analysed in a primarily inductive way, in which the codes were driven by the content of the data, rather than applying a coding framework based on prior theories or ideas. However, codes were then organised into themes with the two research questions in mind, rather than a purely inductive way. The analysis was approached from a realist perspective (reporting an assumed reality present in the data [50]) and codes were developed at a semantic level, by examining the surface meeting of the data.

The first step was familiarisation with the data through listening to the interviews whilst reading the transcripts, noting any initial reflections in the journal. This was followed by inductive coding of the data, giving equal attention to each interview. Coding was initially carried out on each transcript before being transferred to copies of the transcripts stored in Nvivo (v11). The latter was then used to organise (rename, combine, and divide) the codes. The final codes were printed and grouped together on paper under initial potential themes. The themes were checked against the interview transcripts, reflexive journal and field notes and discussed with two independent qualitative researchers to refine them and ensure they remained close to the original data. They were then discussed with the research team who defined and named the final themes. The themes were then incorporated into a written narrative evidenced with data extracts.

### Mixed methods integration and analysis

The intent of integration in a convergent design is “to develop results and interpretations that expand understanding, are comprehensive, and are validated and confirmed” (Creswell & Plano Clark, p.221 [44]). Integration occurred at both the methods level, through basing interview questions on the topics of three questionnaire scales, and at the results level, through comparing interview and questionnaire data in a process known as *merging* [51]. Merging was conducting by CN and co-authors RB, JA and BR. When comparing the quantitative and qualitative results, we examined four possible outcomes [52]: 1) *Confirmation*, when the quantitative and qualitative findings lead to the same interpretation 2) *Complementarity*, when the two sets of data show different, non-conflicting conclusions 3) *Expansion*, when the datasets provide a central overlapping theme and a broader non-overlapping interpretation 4) *Discordance*, when the two datasets lead to conflicting interpretations. The outcomes are presented in a cross-tabulation format [53] to illustrate how the findings compare.

## Results

### Quantitative results

A total of 167 low vision practitioners were invited to take part in the online questionnaire, of which 122 (73.1%) completed it and consented for their responses to be used for research purposes. Table 1. summarises the background characteristics of the participants and their overall scores on the three questionnaire scales. The three questionnaire scales were Rasch analysed and, after iteratively removing mis-fitting items and those displaying DIF, they displayed adequate psychometric properties, with ordered response thresholds, no mis-fitting items or item bias, and minimal evidence of multidimensionality (See Additional file 2 – Rasch Analysis Methodology & Results).

**Table 1:** Summary of the background characteristics and overall scores of participants who completed the questionnaire

Characteristic/Score	*N* = 122
Age (years), Median (IQR)	44.0 (38.0–54.0)
Data Missing, *n* (%)	1 (0.8)
Gender, *n* (%)	
Male	50 (41.0)
Female	72 (59.0)
Professional Background, *n* (%)	
Optometrist or Ophthalmic medical practitioner	113 (92.6)
Dispensing optician	8 (6.6)
Data Missing	1 (0.8)
Primary Place of Work, *n* (%)	
Independent practice working with others	58 (47.5)
Independent practice working on own	37 (30.3)
Multiple practice working with others	19 (15.6)
Multiple practice working on own	3 (2.5)
Other	5 (4.1)
Type of Assessments, *n* (%)	
Practice based	73 (59.8)
Domiciliary	4 (3.3)
A mixture of both	45 (36.9)
Time since professional registration (years) Median (IQR)	21.0 (14.0–42.0)
Time employed in eye care services (years) Median (IQR)	21.0 (14.0–31.0)
Time employed as LVSW practitioner (years) Median (IQR)	9.0 (6.0–10.0)
Average number of people with low vision seen each month, Median (IQR)	5.0 (4.0–10.0)
Average time spent with person with low vision (mins) *n* (%)	
less than 10	0 (0.0)
11–20	0 (0.0)
21–30	8 (6.6)
31–40	29 (23.8)
41–50	47 (38.5)
51–60	32 (26.2)
more than 60	6 (4.9)
Previous training on depression, *n* (%)	
Yes	7 (5.7)
No	115 (94.3)
Part B: Action in practice scale Rasched score, Median (IQR)	−1.710 (−3.430, −0.150)
Part C: Confidence scale Rasched score, Median (IQR)	−1.820 (− 3.460, 0.170)
Part D: Barriers scale Rasched score, Median (IQR)	−0.750 (− 1.450, − 0.070)

### Research Q1: current practice around identifying and responding to depression

Data from the LVSW record cards indicated that of 162 practitioners who completed assessments between 1st July and 31st December 2017, 29 (17.9%) recorded risk of depression for at least one patient. In the online questionnaire, 40 (32.8%) practitioners indicated that they currently aimed to identify possible depression in patients with low vision. The majority did not use a screening tool to identify depression, with 107 (87.7%) selecting ‘never/rarely’, 8 (6.6%) ‘less than half the time’, 7 (5.7%) ‘more than half the time’ and 0 ‘always/almost always’. When acting in response to suspected depression, practitioners were most likely to discuss the patient’s feelings with them and least likely to provide a referral to mental health services (see Additional file 3: Figure S1. for responses to all action in practice scale items).

### Research Q2: characteristics linked to current practice

We examined whether practitioners’ current practice was related to their demographic or work-related characteristics, confidence score or barriers score. Practitioners with a longer time since professional registration or those performing a mixture of assessment types were less likely to report that they aimed to identify depression (Table 2). In contrast, those with a higher confidence score in working with low vision patients with depression and those in a dispensing optician role were more likely to report aiming to identify depression.

**Table 2:** Stepwise multivariate logistic regression to determine characteristics related to identifying depression (Reference: No)

Variable	*N*	OR	95% CI	*p*-value
Time since professional registration (years)	120	0.957	0.919 to 0.998	0.040
Professional Background: Dispensing optician (vs Optometrist or Ophthalmic medical practitioner)		6.312	1.130 to 35.271	0.036
Type of Assessments: A mixture of both (vs Practice based or Domiciliary)		0.331	0.124 to 0.879	0.026
Confidence total score		1.407	1.148 to 1.726	0.001

Log likelihood = −60.420; AIC = 130.841; BIC = 144.778; adjusted (pseudo) *r*^2^ = 0.1935

With respect to the likelihood of taking further action when depression is suspected, practitioners who have been employed for longer as a LVSW practitioner or who scored higher on the perceived barriers scale were less likely to report taking action (Table 3.). Those who scored higher on the confidence scale or those in the role of a dispensing optician were more likely to act in response to suspected depression. Despite relatively low adjusted r^2^ values, indicating a weak overall relationship, the stepwise procedure still found multiple statistically significant predictors. For item responses on the confidence scale items see Additional file 4: Figure S2. and for the barriers scale items see Additional file 5: Figure S3.

**Table 3:** Stepwise multivariate linear regression to determine characteristics related to action taken in response to depression

Variable	*N*	Effect Size	95% CI	*p*-value
Professional Background: Dispensing optician (vs Optometrist or Ophthalmic medical practitioner)	120	1.992	0.538 to 3.445	0.008
Time employed as LVSW practitioner (years)		−0.155	−0.245 to − 0.064	0.001
Confidence total score		0.228	0.081 to 0.376	0.003
Barriers total score		−0.573	− 0.903 to − 0.244	0.001

Log likelihood = − 246.356; AIC =502.712; BIC = 516.650; adjusted *r*^2^ = 0.3539

### Qualitative results

Of the 12 participants (6 male) interviewed, nine were optometrists and three were dispensing opticians who worked either in independent or multiple practices or both and performed a mixture of practice based, domiciliary or both types of assessments. The length of time that they had worked in eye care services and in the LVSW ranged from 2.5 to 38 years, and 1–11 years, respectively. The number of low vision patients seen each month ranged widely, from 2 to 55 and the length of time spent in consultation with a patient ranged from 31 to 60+ minutes. We present a brief introduction to the interview findings before addressing the two research questions.

From the sample of 12 practitioners, 10 reported some level of personal experience of depression, either experienced by themselves or by close family members or friends. They understood depression could be *“pretty debilitating and pretty horrible for people”* (P01), *“an awful sort of blackness which descends on you”* (P08) and described several aspects of the disorder including emotional (e.g. sadness), cognitive (e.g. low motivation) and behavioural (e.g. reduced activity). Of the two remaining practitioners, one described depression as having low mood and the other reported *“not [knowing] a great deal to be honest”* (P06). Seven practitioners referred to their personal experience, or lack of it, as having an impact on their work with low vision patients:

*“Because I don’t have so much knowledge and experience of depression myself, because like I said I’ve not dealt with it first-hand … perhaps that’s why I find it limiting, personally, talking about it [with patients].”* (P10)

Due to the current lack of requirement for the LVSW practitioners to address depression, the practitioners expressed varying views as to whether doing so is part of their role. Two practitioners did not consider it to be their responsibility, and perceived depression to fall under the remit of the General Practitioner (GP). Others referred to continuously expanding roles and believed it should be part of their assessment, especially when mental health difficulties were vision related or affected rehabilitation.

*“ … .. it’s a multidisciplinary role, we’re not just doing what, what magnify can you see through … yeah I think there’s a definite holistic side to low vision as well as just being clinical about it.”* (P02)

In addressing the research questions, three themes were identified: 1) Depression is an understandable response to vision loss 2) Patients themselves are a barrier to addressing depression 3) Practitioners lack confidence in their knowledge and skills to address depression.

#### Theme 1: depression is an understandable response to vision loss

The majority of practitioners view poor health, physical limitations, old age and vision impairment as particular risk factors for depression, and the prevalence of depression in their patients (who typically meet most of these criteria) is considered to be high. The majority view depression as an understandable response to vision loss, with some going even further, suggesting it is an inevitable consequence:

*“It’s just part of low vision, which is almost assume they’re going to be depressed ‘cause they’ve lost their eye sight, it’s just how depressed is the thing or how unhappy.”* (P04)

Depression is considered more likely in those with recent or sudden vision loss, and those not able to accept their eye condition:

*“I think some of the kind of longer standing erm, low vision patients they, they’re kind of a bit more accepting of it, so I don’t think they’re too depressed”* (P06)

##### Link between low vision and depression

Practitioners shared their theories on the link between low vision and depression. Common perceptions are that depression results from the activity limitations and loss of independence caused by failing sight, which in turn can lead to loneliness and isolation:

*“I think a lot of the time the reason people get depression with low vision, is they can't do things they used to before. That's very difficult, life changes. And … I think the reason that I say isolation is a big issue, is because they have a lot of activity limitations.”* (P07)

Those who believe depression to result largely from activity limitation perceive their core role of enhancing visual function and promoting independence will have a direct positive impact on mood. Hence, they focus on practical solutions, such as advising on the use of coloured chopping boards to help with meal preparation, referring to social services for mobility training or prescribing aids to help with hobbies such as reading:

*“I always try to be optimistic and say, oh look you’ll be able to be back reading again and you’ll be able to go to the library and you gets lots of books … ..I don’t really say this’ll make you feel better, I suppose that’s just ‘cause I assume it does … … Erm, I just assume that being able to see a bit better will help [with the depression]”* (P04).

To reduce loneliness and isolation, practitioners commonly “signpost” (direct) patients to support groups, clubs and charities for the visually impaired, which they perceive to have a positive impact. One practitioner talked about a local bowls club for the visually impaired:

*“..it's a group of about four or five of them, who've now become very good friends, and who were sort of individual you know, 40 year old men, on their own, who'd lost their vision. And now … .. life has completely changed, because they have got that social aspect, you know.”* (P09)

#### Theme 2: patients themselves are a barrier to addressing depression

##### Patients are reluctant to discuss depression

Ten practitioners perceive ‘the patient themselves’ to be a significant barrier to addressing depression in low vision assessments. These individuals, in addition to one further practitioner, expressed the opinion that patients are commonly unwilling to discuss their mental health, thus hindering the identification of depression:

*“The biggest one (barrier) for me um … .I would say it’s probably trying to get the patient to open up”* (P02)

Practitioners feel this reluctance is due to the societal stigma associated with depression and that having depression could be perceived as a sign of weakness or inferiority:

*“There is a general taboo about discussing mental illness within society as a whole isn’t it? People with mental illness tend to be looked down on. Er, they’re considered to be inferior and unless we can get over that then I think we’re on a hard road.”* (P05)

This is considered to be particularly evident for armed forces veterans and in the older population, who constitute the majority of individuals with low vision.

*“I would say from my experience … .. so low vision patients that are older, which does tend to make most of your low vision database anyway, they tend to be um, very unfamiliar and … .I would say less welcome of mental health issues”* (P07)

Practitioners believe that patients may fear the possible consequences of admitting that they have depression, for example, being viewed as suicidal or unable to cope, being forced into residential care or even institutionalised:

*“Erm, yeah, and fear of what family are going to think, are they going to put me in a home thinking that I’m depressed and I can’t cope and I can’t live on my own anymore.”* (P11, giving a patient perspective)

Given the perceived unwillingness of patients to discuss their mental health, practitioners expressed a reluctance to initiate a conversation about depression:

*“If they were happy to talk about it, I'd be very happy to talk about it … I would say I have more of a reservation on bringing it up or actively talking about it, if the person has not shown me signs they'd be happy to talk about it themselves.”* (P08)

Nine practitioners reported trying to recognise whether a patient was affected by depression. However, because of the patients’ perceived unwillingness to discuss the topic, none of the practitioners use a validated screening tool or ask direct questions about depression. Rather, they rely on a ‘getting a general feel’ or ‘impression’ for the patient’s mood by considering their demeanour and weighing up the conversation.

*“I don’t kind a have a generic question that I would put in every single Low Vision Assessment I do, to kind of say “Do you suffer with depression, yes or no?”, in that kind of screening sense. … .I kind of just weigh up the conversation as it goes, and what I’m absorbing about that person and what they’re telling me really.”* (P02)

‘Red flags’ or ‘warning signs’ that practitioners look for include an abrupt or rude demeanour, appearing disorganised, lack of motivation or engagement to try any aids, reduced interest in hobbies or living alone/being isolated:

*“Specifically if they state that um that they’re not interested in, in it [their hobbies] anymore, then I, I think that sets alarm bells ringing yes”* (P08)

##### Not expecting to discuss with their optometrist

Whilst some practitioners view it within their remit to consider the mental health of their patients, they expressed concerns that the patient would not expect this in a low vision assessment. Three individuals held a view that patients do not consider the role of practitioners to incorporate the management of depression, either because they do not perceive practitioners as healthcare professionals or because they believe the practitioners’ role to be limited to correcting sight:

*“You know, at the end of the day they have just sort of in their mind come in to get some magnifying glasses, um so they might be a bit kind of blind-sided a bit if you start going down that sort of route really.“* (P03)

Therefore, practitioners fear that opening a conversation about depression would be perceived as ‘nosey’, inappropriate and intrusive, particularly for older patients, and could damage their working relationship or deter the patient from returning in future:

*“ … patients can get quite defensive and difficult and what you don’t want is to … close the door when actually we could be quite helpful to them. And then not want to go and see the optician because the optician’s going to get the white coat, er, get the straight jacket out and send me away and that’s not, obviously the idea, but it’s, I think what people might think, some people.”* (P01)

The common experience of practitioners is that on the occasions they had asked about their patients’ mood, the conversation was usually curtailed:

*“ … people very quickly close off and, and don’t want you to know that things aren’t okay and they’re like “No, no I’m fine, I’m coping with that, I’m all sorted thank you.”* (P02)

##### Patients reluctant for formal help

Practitioners also perceive that patients generally decline support for their mental health, reflecting the wider reluctance of the older generation to accept help. They reported that patients sometimes seem defeated, ‘want to be left alone’ and do not want to be prescribed more medication.

“*…*. *that’s the sort of feeling that you get from them is that they’re sort of reluctant to, to take on board anything that might help them, um, it’s sort of almost defeated, that kind of thing really.”* (P03).

In such instances, practitioners feel limited in their ability to help. The majority cited anti-depressants and/or therapy as the most recognised forms of intervention for depression and acknowledged these were available via the GP. However, they were uncertain about how to approach gaining consent to make a referral to the GP. Some reported approaching the discussion in a roundabout manner:

“*… I try to kind of say to them in a matter of “How would they feel about getting a bit more support in the area they feel they’re struggling with?” Rather than me going “I think you’re really low, you need a referral.”* (P02).

They reported that such suggestions were often dismissed and did not result in GP referral.

#### Theme 3: practitioners lack confidence in their knowledge and skills to address depression

Throughout the interviews, 10 practitioners expressed a lack of confidence in their knowledge and skills in working with people with depression.

##### Lack of confidence in own knowledge

During the interviews practitioners were frequently hesitant and moderated their opinions about depression with terms such as “I think”, “I guess” or “I assume”. Some practitioners cited their lack of knowledge as a barrier to their ability to correctly recognise depression. They believe it differs between individuals and acknowledged that some could hide it well, thus making it easy to miss:

*“ … because it affects people differently on different days as well you, you could have someone that came in you know … .completely normal and you wouldn’t think anything was wrong, and but it’s almost (pause) yeah, it’s so hard.”* (P12)

Conversely, the practitioners shared concerns about making an incorrect judgement about a patient who was mentally well, which may cause unnecessary distress:

*“What if I make the wrong call? What if I, you know, upset either the patient or cause some unnecessary investigation when actually there’s nothing to warrant concern.”* (P11)

As a consequence, they are more likely to refer ‘obvious’ and/or ‘serious’ cases of depression, rather than potentially ‘incorrectly’ referring borderline or less obvious cases:

*“ … if I have done it [a GP referral] … it’s been when it’s been quite serious and quite obvious and it’s been, you know, a way of avoiding them causing harm to themselves or to others. So, it’s always been a serious sort of referral and not a, not if somebody’s feeling as I would call it, low or down.”* (P10)

A lack of knowledge of what the GP might be able to offer the patients also lead to a reluctance to refer to them, and to rely on support groups instead:

*“I’m not sure what services my GP would be able to offer um the patient and you always think along the lines of counselling and other charities and support groups but really I, I don’t know is, is the honest answer.”* (P10)

For some, a lack of knowledge about appropriate referral pathways for patient with suspected depression meant they were unwilling to instigate any conversation about depression:

*“So, I probably won’t have that direct conversation [about depression], as I don’t really know what I’m gonna do with the information once I get it … . I don’t know is, is the honest answer, err who to refer the patient to.” (P10)*

##### Lack of confidence in communication skills

Lack of confidence in their communication skills is also an issue:

*Interviewer: “ … what do you think is the single biggest barrier to this work?”*

*Practitioner: “Um, I think it’s my awkwardness at raising … the question [about depression].”* (P08)

The majority of practitioners are cautious of discussing suspected depression with patients. A common fear is that by initiating a conversation about mental health, for which they do not feel qualified or trained, they might somehow ‘do more harm than good’:

*“I think that’s it … I don’t know enough about it and I’m not qualified to do it so, erm … I don’t want to do the wrong thing and I don’t want to say the wrong thing to people ‘cause people might be quite sensitive to me saying the wrong thing and, er … It could do more harm than good, that’s the worry, it’s doing more harm than good … So, erm, that’s, I think the be all and end all of it I think.”* (P01)

Perceived potential harms include causing embarrassment, discomfort or upset.

*“Certainly with older patients some of them are quite private, they’ve got a lot of privacy, got to be very careful, what you say um and yeah I think maybe for the majority of practitioners, if, if you haven’t had training, it’s probably something we’re not that confident in addressing in fear of upsetting a patient.”* (P02)

Practitioners also had concerns of causing a more detrimental impact on the patient’s mental health, for example, by ‘pushing them over the edge’:

*“ … it’s knowing how to do that [talk about depression] safely … ..without endangering the mental health of your patients, but I think that’s perhaps why a lot of people are frightened to step in … erm, because you don’t know what the patient’s going to feel after they’ve left you. Are they in a better place or have you inadvertently pushed them into a darker place?”* (P05)

Several practitioners compared initiating a conversation about depression to ‘opening a can of worms’ that they lacked the confidence to contain. They perceive that appearing obviously unprepared or unqualified for the discussion might cause the patient annoyance and ‘close the door’ to them returning for follow up:

*“ … it’s the follow up questions and why do you think I’m depressed, I’m not depressed and then making him upset and if the patient then gets, erm, patients can get very, very defensive and seeing as I had a good rapport with him, I don’t want to spoil that ‘cause I want to see him again.”* (P01)

Practitioners also expressed concerns about lacking the skills to open and close a discussion within the time allocated for a low vision assessment, and that this could impact on the running of the clinic.

##### Training and protocols required

Whilst there were some opposing opinions as to whether it was within the practitioners remit to address depression in low vision patients, the general sentiment was that *“If I don’t, then who will?”* (P03). However, the majority clearly expressed a need for training and protocols in order to feel confident to incorporate depression screening and management pathways into standard low vision assessments:

*“It’s definitely an area that we need more training in, there’s no doubt about that.”* (P03)

*“So, what it would take is for someone to instruct and to say ‘Okay this is what you now need to be doing as part of your low vision assessments, refer these patients that fit into these categories for these sorts of referrals … because they’ll receive this sort of help’, um so if I had some clarity and instruction and guidance, I think I would do it.”* (P10)

### Mixed methods results

The quantitative and qualitative findings were merged and compared for confirmation, complementarity, expansion and discordance. Three key findings around the use of screening tools and influences on current practice resulted in expansion, with the interviews expanding and explaining the results shown in the survey data. There was one instance of discordance between the two datasets, around the percentage of practitioners aiming to identify depression. Reasons for this are considered in the discussion. None of the results from the two datasets were considered to result in confirmation or complementarity Table 4.

**Table 4:** Outcomes from merging the questionnaire, record card and interview results

MERGING OF RESULTS		OUTCOME
Quantitative	Qualitative	
Q1: Current practice		
Identification of depression		
The quantitative data suggest only a minority of practitioners currently try to identify depression in low vision assessments.	The majority of practitioners interviewed reported trying to identify if a patient was depressed.	Discordance
Practitioners do not use a screening tool		
On the questionnaire, a substantial majority (88%) of practitioners reported not using a screening tool to identify depression.	None of the practitioners interviewed used a screening tool. They revealed that: 1) they did not know what screening questions to ask and 2) wanted to avoid broaching the subject of depression directly with the patients, to avoid causing harm. Instead they considered the patient’s demeanour and weighed up the conversation, looking for ‘red flags’ which gave them a ‘general feeling’ or ‘impression’ that the patient might be depressed.	Expansion
Q2: Influences on current practice		
Confidence level		
Reported level of confidence was associated with intention to try to identify depression and likelihood of taking any action in response to suspected depression.	‘Practitioners lack confidence in their knowledge and skills to address depression’ was a key theme identified in the qualitative analysis and was shown to affect practice. Most lacked confidence in their communication skills and were reluctant to ask about possible depression for fear they might cause ‘more harm than good’. Therefore, when they suspected depression, they approached the discussion about support options in a roundabout manner and found it difficult to gain consent for referral, thus limiting the action they could take. Many also expressed a lack of confidence in their knowledge in recognising depression, which influenced their response with regard to GP referrals – only those with ‘serious’ or ‘obvious’ depression were referred.	Expansion
Perceived barriers		
Practitioners who perceived more barriers to working with people with depression were less likely to action in response to suspected depression.	‘Patient themselves are a barrier to addressing depression’ was a key theme. Practitioners suggested patients were unwilling to discuss their mental health and frequently declined support, leaving the practitioner with limited options for responding to suspected depression. Other barriers to taking action included their lack of knowledge of suitable referral pathways and what a General Practitioner might be able to offer.	Expansion

## Discussion

The aim of this study was to understand community-based low vision practitioners’ current practice around identifying and responding to depression in their patients, and to examine the characteristics and beliefs linked to their practice. Despite the high prevalence of depressive symptoms in patients attending the service, only one third of practitioners who completed an anonymous online questionnaire reported that they currently aim to identify depression in their patients. Even fewer had ticked the box on the service record card for at least one patient, to indicate a possible at risk of depression. This is understandable, given practitioners are not yet formally required to consider depression as part of the assessment. In terms of methods for identifying depression, only a small minority of practitioners use a validated screening tool. Those who reported feeling more confident working with people with depression were more likely to both identify depression and take action to manage it, whilst those who perceived more barriers were less likely to take any action. Few personal or work-related characteristics were associated with practice: dispensing opticians were more likely to identify and act on depression, whilst those who had been registered for longer as an eye care professional, those performing both home and practice based visits and those worker for longer in the LVSW were less likely to address depression. We note that the confidence intervals for the logistic regression finding regarding dispensing opticians were wide, possibly due to the small number of this profession in the study (and the service). Therefore this finding should be interpreted with caution.

The interviews revealed that those who do try to identify depression rely on cues from, and conversation with, the patient to get a general feeling about whether someone may be depressed. They consciously avoid direct questions and conversations about depression, primarily because they believe patients to be reluctant to discuss their mental health, particularly with their optometrist or optician. They attribute this reluctance to the stigma associated with the condition which they believe to be worse for older people, and perhaps because they do not view low vision practitioners as health care professionals. Practitioners generally lack confidence in their communication skills around depression and fear that by talking about possible depression, they could be perceived as being nosey or inappropriate, upset the patient and do more harm than good. Along with perceived patient reluctance to seek treatment, this makes it difficult to discuss support options for suspected depression. Practitioners reported approaching such conversations in a roundabout manner which rarely leads to any action. A lack of confidence in their knowledge about mental health was also seen as a barrier to addressing depression. Some were unsure how to correctly identify depression, which led to GP referrals only for the most serious and hence obvious cases. Practitioners were also unsure of what the GP had to offer more moderate cases and therefore were reluctant to refer to them. They were more confident to refer to social services and support clubs which they thought could help to overcome the activity limitations, social isolation and loneliness caused by vision loss. They viewed depression as an understandable, almost inevitable, response to low vision and thought enhancing visual function could improve mood by helping people to re-engage with activities.

The mixed methods analysis revealed that the qualitative dataset largely overlapped with and expanded the data collected in the questionnaires, providing insights into the questionnaire responses. There was one instance of discordance: the majority of practitioners interviewed reported trying to identify depression, compared to only a third on the questionnaire. This discrepancy may be for a number of reasons. Firstly, it could be due to social desirability. The interviewees may have told the interviewer what they thought was the ‘correct’ answer ie. they do try to identify depression. Alternatively, it may have been influenced by the time available to interview participants to reflect on and discuss their practice with the interviewer. For example, two practitioners initially said they did not try to identify it, before changing their mind and realising they did so on an informal basis.

Our findings corroborate similar research with eye care professionals and rehabilitation workers in Australia. In a quantitative study, 40% of practitioners reported aiming to identify depression, only 4% used a screening tool and confidence and perception of barriers were linked to likelihood of identifying and acting on depression [38]. In focus groups, tertiary eye care professionals also reported using behaviour and demeanour to recognise depression, referred patients to support groups and felt there was only a clear referral pathway for serious cases [37]. Perceived barriers included patient reluctance to discuss depression due to stigma, confusion about their role and system barriers such as time and lack of available private space. Our work has expanded upon these previous findings, demonstrating similar practices and concerns across continents, eye care settings and job roles.

Moreover, our findings echo those from the wider chronic health and older adult literature. Primary care professionals working with the elderly, and health care professionals working with people with diabetes and chronic heart disease, hold the same view as the low vision practitioners: that depression is understandable, justifiable or even inevitable, a normal response to the patient’s situation rather than a disorder [23, 54]. This perspective was also shared by both the elderly and chronically ill patients themselves, and Burroughs et al. concluded [23], it leads to ‘therapeutic nihilism’ [55], a lack of belief in potential treatments, particularly within the biomedical health service model [56]. This may explain why low vision patients are perceived to be reluctant to accept a GP referral and why practitioners signpost to social services and support groups for social engagement instead.

Previous work also confirms our other two key themes. A UK based ethnographic study of general practices revealed many patients with chronic heart disease and diabetes did not understand why they were being asked about depression as part of routine case screening and sometimes gave defensive or defiant answers [34]. The patients were concerned that they were being perceived as someone who could not cope. This is in line with the low vision practitioners’ views that patients are reluctant to discuss their mental health, for fear of being perceived as weak, and therefore their reticence to address depression directly with a patient. When asked about discussing and diagnosing depression in late-life, none of the GPs in a qualitative study [23] reported using formal schedules but instead used their ‘intuition’ and own style of questioning. They acknowledged that making a diagnosis was difficult. Similarly, health care professionals working with people with diabetes and chronic heart preferred to incorporate subtle methods of identifying depression into their assessment, particularly with patients with whom they had a relationships [23, 54].

In terms of confidence in working with people with chronic and depression, primary care practitioners revealed they did not feel confident in how to approach screening and used the term ‘can of worms’ to describe their own and patients’ discomfort with case finding for depression [34]. Many felt it was their responsibility to deal the problem, rather than advise the patient to visit the GP, which led to an emotional burden. Nurses working with older adults also reported lacking the expertise to discuss mental health and had no protocols to assist in identifying or managing an elderly patient with depression [23].

### Implications for practice

The majority of practitioners in the LVSW do not yet routinely assess low vision patients for depression and feel they lack the knowledge and skills to do so effectively. Before implementing routine screening for depression into this or any chronic illness service, practitioners need to be fully prepared [56] and practitioners themselves expressed a need for training. Firstly, they require the knowledge to confidently identify possible cases of depression, including information on key signs and symptoms. Use of a simple validated screening tool such as the two Whooley questions [30] may improve rates of case finding and practitioners’ confidence in a ‘correct’ assessment, over relying solely on intuition. However, this would entail addressing depression directly, which is something practitioners currently avoid. Therefore, a key element of a training program would also need to cover communication skills including how to initiate and contain a conversation about depression and how to respond to emotion. Screening by itself does not improve patient outcomes [32]. Hence, any service needs to establish a clear referral pathway. For the LVSW, it has been established that referral to the GP is part of the service protocol. To feel confident with this recommendation, practitioners would also need advice on negotiating patient consent and writing the referral letter. Trainers would also need to challenge practitioners’ beliefs that depression is inevitable and patients will not benefit from treatment, for referrals to occur. Similarly, the concerns about patient reluctance to acknowledge their depression would need to be addressed. Perhaps presenting screening as a normal and routine part of care may help reduce feelings of shame and give patients ‘permission’ to discuss depression [56].

### Strengths and limitations

We used a mixed methods design to examine clinical practice from both a quantitative and qualitative perspective. The qualitative results largely confirmed and expanded the quantitative results, adding credibility to the study findings. There was one instance of discordance which highlights the importance of using both questionnaire and interview approaches to overcome potential limitations of using a single method [43]. Rasch analysis was used to optimise the psychometric properties of the quantitative questionnaire scales, transform ordinal responses into interval-level measurements and demonstrate the reliability of the questionnaires.

The study benefited from a high response rate to the questionnaires, enhancing the generalisability of the findings. Data was largely complete, with missing data only in two cases. The thematic analysis was rigorous, thereby enhancing the trustworthiness of the qualitative findings. Overall, the study expands previous research with eye care practitioners by including the perspectives of optometrists and dispensing opticians, examining community based low vision rehabilitation and using a mixed methods approach. The main limitation is that, whilst the response rate was high, we do not have any information on those who did not complete the questionnaire. Therefore, there may be a risk of bias as the non-completers may be systematically different from those that completed the questionnaire. It is feasible that those who took part are more interested in mental health and therefore more motivated to try to identify and record risk of depression. In addition, it would have been preferable for a researcher independent of the LVSW to have invited practitioners to take part, however, it was only logistically possible for the Clinical Lead to do so in this study.

## Conclusions

Our findings indicate that, despite the high prevalence of depression in people with low vision, community-based practitioners do not routinely screen for depression. Those who do try to assess depression rely on their intuition to do so. This leads to lack of confidence in this assessment, and combined with their views that depression is an understandable response to vision loss and that patients are reluctant to accept help, means they rarely refer a patient to the GP for further assessment and support. These findings reflect those found in the wider chronic health and older adult literature. Before introducing routine depression screening and referral into this or any service, practitioners need training to improve their knowledge and communication skills, along with clear service protocols. Given the ageing population and their greater susceptibility to reduced mobility, chronic pain, frailty or other health problems leading to poorer mental health [57], embedding training in undergraduate programs is timely for all future primary and community care health professionals.

## Supplementary information

**Additional file 1.** Study Questionnaire. Word document (.doc). A copy of the questionnaire used in this study.

**Additional file 2. **Rasch Analysis Methodology and Results.. Details the Rasch analysis methodology used and the outcomes, including **Table S1.** The psychometric properties of the three scales utilised in the study.

**Additional file 3. **Responses to action in practice scale. **Figure S1.** indicates the responses to all action in practice scale items.

**Additional file 4. **Responses to confidence scale. **Figure S2.** indicates responses to all confidence scale items.

**Additional file 5. **Responses to barriers scale. **Figure S3.** indicates the responses to all barrier scale items.

## Data Availability

The datasets used and analysed during the current study are available from the corresponding author on reasonable request.

## Abbreviations

ECP: Eye care professional
GP: General Practitioner
LVSW: Low Vision Service Wales
OR: Odds ratio
