# Supplementary material for: How do community-based eye care practitioners approach depression in patients with low vision? A mixed methods study
Source: BMC Psychiatry. 2019 Dec 30;19:426. doi: 10.1186/s12888-019-2387-x (PMC6937690; doi:10.1186/s12888-019-2387-x)
Supplement: Supplementary file 1 — Additional file 1. Study Questionnaire. Word document (.doc). A copy of the questionnaire used in this study. [file 12888_2019_2387_MOESM1_ESM.doc]

**Additional File 1 – Study Questionnaire**

**Part A: Professional background**

1. Gender:

□ Male □ Female

1. What is your professional background?

□ Optometrist or Ophthalmic Medical Practitioner

□ Dispensing Optician

1. What year did you gain full GOC registration? _ _ _ _
2. What is your primary place of work?

□ Independent practice working with others

□ Independent practice working on own

□ Multiple practices working with others

□ Multiple practices working on own

□ Hospital

□ Other *(please specify)*

1. What type of assessments do you conduct?

□ Practice based

□ Domiciliary

□ A mixture of both

1. Approximately how long have you been working **in eye care services**?

_____ year(s)

1. Approximately how long have you been working as a Low Vision Service Wales accredited practitioner?

_____ year(s)

1. In your current position(s), please estimate approximately how many people with vision impairment you see each month on average _____
2. In your current position(s), please estimate approximately the average time (minutes per assessment) that you spend with each person with vision impairment?

less than 10mins 11-20mins 21-30mins 31-40mins 41-50mins 51-60mins more than 60min

1. Have you previously had any specific training related to depression?

□ Yes □ No

**Part B: Current practice**

1. Do you aim to identify possible depression as part of patient management for patients with a visual impairment?

□ Yes □ No

1. When assessing patients with a vision impairment, how often do you use a depression screening tool (for example, the two questions from the NICE guidelines on depression, or a questionnaire)?

□ Never/rarely □ Less than half the time □ More than half the time

□ Always/Almost always

1. **If you suspect a patient with vision impairment is depressed,** how often are you likely to do each of the following actions:

|  | **Never**  **/rarely** | **Sometimes** | **Often** |
| --- | --- | --- | --- |
| Discuss their feelings with them | 1 | 2 | 3 |
| Provide the patient with written resources about depression and support options | 1 | 2 | 3 |
| Discuss with family members if possible | 1 | 2 | 3 |
| Discuss referral with the patient | 1 | 2 | 3 |
| Provide a referral to a self-help or support group | 1 | 2 | 3 |
| Refer the patient to their GP | 1 | 2 | 3 |
| Provide a referral to a mental health service such  as counselling or a psychologist *(please specify)* ______________ | 1 | 2 | 3 |
| Other *(please specify):* ____________________ | 1 | 2 | 3 |

**Part C: Confidence in working with patients with low vision and depression**

Tick the number that best describes how confident you feel in **working with patients with low vision and depression**.

|  | Not confident | Slightly confident | **Mostly confident** | **Very confident** |
| --- | --- | --- | --- | --- |
| 1. In **asking** patients with vision impairment about their feelings or mood, I feel … | 1 | 2 | 3 | 4 |
| 1. In **listening** to patients with vision impairment talk about their feelings or mood, I feel … | 1 | 2 | 3 | 4 |
| 1. In being able to **recognise** that a patient with vision impairment might be depressed, I feel ... | 1 | 2 | 3 | 4 |
| 1. In knowing which **signs** to look for to tell if a patient with vision impairment might be depressed, I feel ... | 1 | 2 | 3 | 4 |
| 1. In knowing if a patient might have depression or is just **dissatisfied with their current situation**, I feel … | 1 | 2 | 3 | 4 |
| 1. In discussing my concerns about possible depression with a patient’s **family members**, I feel … | 1 | 2 | 3 | 4 |
| 1. In **providing education** on the link between vision impairment and depression, I feel … | 1 | 2 | 3 | 4 |
| 1. In **providing education** on possible **treatment strategies** for depression, I feel … | 1 | 2 | 3 | 4 |
| 1. In **directing** a patient who might be depressed to appropriate services or agencies, I feel … | 1 | 2 | 3 | 4 |
| 1. In passing on my concerns about possible depression to a patient’s **GP**, I feel … | 1 | 2 | 3 | 4 |
| 1. In passing on my concerns about possible depression **to vision rehabilitation agencies**, I feel … | 1 | 2 | 3 | 4 |

**Part D: Barriers to working with patients with low vision and depression**

Please circle a number to indicate whether you disagree or agree with each statement, in relation to the **patients with low vision that you provide care for**:

|  |  | Strongly Disagree | Somewhat Disagree | Somewhat Agree | Strongly Agree |
| --- | --- | --- | --- | --- | --- |
| 1 | I don’t have enough **time** to talk with patients to tell if they might be depressed. | 1 | 2 | 3 | 4 |
| 2 | My **high** **workload** makes it difficult to know if a patient might be depressed. | 1 | 2 | 3 | 4 |
| 3 | The absence of **standard procedures** to follow in my workplace when I suspect a patient is depressed means they may not always receive the best management for depression. | 1 | 2 | 3 | 4 |
| 4 | Depression is not addressed because the **environment** in which I work is not suitable for private discussions about emotional well-being. | 1 | 2 | 3 | 4 |
| 5 | **Family members** attending the consultation means it is difficult to have an open discussion about depression with the patient. | 1 | 2 | 3 | 4 |
| 6 | **Patients’ reluctance** to discuss how they feel makes it difficult to tell if they might be depressed. | 1 | 2 | 3 | 4 |
| 7 | The possibility of depression is not explored because I need to **protect myself** from being involved with patients’ emotional problems. | 1 | 2 | 3 | 4 |
| 8 | Depression does not receive enough attention, because **my role** is related to patients’ eye health rather than emotional well-being. | 1 | 2 | 3 | 4 |
| 9 | My limited **knowledge** of depression means that patients may not always receive the best management for depression. | 1 | 2 | 3 | 4 |
| 10 | **Language and/or cultural** barriers make it difficult to discuss depression with patients. | 1 | 2 | 3 | 4 |
| 11 | My **supervisor/team leader/ophthalmologist** does not believe that detecting depression is part of my role at work. | 1 | 2 | 3 | 4 |
| 12 | My poor **knowledge of what to do** if a patient could be depressed means that they may not always receive the best management. | 1 | 2 | 3 | 4 |
| 13 | My **supervisor/team leader/ophthalmologist** is reluctant to listen to my concerns that a patient might be depressed. | 1 | 2 | 3 | 4 |
