# Supplementary material for: How do community-based eye care practitioners approach depression in patients with low vision? A mixed methods study
Source: BMC Psychiatry. 2019 Dec 30;19:426. doi: 10.1186/s12888-019-2387-x (PMC6937690; doi:10.1186/s12888-019-2387-x)
Supplement: Supplementary file 2 — Additional file 2. Rasch Analysis Methodology and Results.. Details the Rasch analysis methodology used and the outcomes, including Table S1. The psychometric properties of the three scales utilised in the study. [file 12888_2019_2387_MOESM2_ESM.docx]

**Additional file 2 - Rasch Analysis Methodology & Results**

**Methodology**

Rasch analysis is a form of Item Response Theory (IRT) in which the ordinal ratings of the questionnaire are transformed to estimates of interval measures (expressed in log of the odds units, or logits). The advantages of IRT over classical test theory (CTT) has been well-documented [1]: in brief, it provides a much richer description of the performance of each item compared to CTT, which is useful during patient-reported outcome measure development, provides greater detail on a measure’s precision, which may vary across different levels of the construct at the item or scale level, as compared to CTT, which uses only a single estimate (e.g. Cronbach’s alpha), and scores estimated using IRT methods are independent of item difficulty as opposed to observed scores using CTT. During Rasch analysis, responses were recoded so that higher scores indicated greater willingness to act in response to perceived depression in low vision patients (“action in practice” scale), greater confidence in working with individuals with concomitant low vision and depression (“confidence” scale) and greater perceived barriers in working with low vision patients with depression (“barriers” scale), respectively. Differential item functioning (DIF), a measure of whether an item measures an ability the same way across different groups, was assessed for age (median split <44 years vs. ≥44 years), gender and whether the practitioner had previously received training in the identification and management of depression.

**Results**

Initially, all three scales displayed poor fit to the Rasch model, with suboptimal precision, evidence of multidimensionality (> 1 construct being assessed) and DIF, as well as misfitting items (see Supplementary Table 1). After iteratively removed misfitting items and those displaying DIF (item 3 from the action in practice scale, item 5, 9 and 11 from the confidence scale, and items 3, 9 and 12 from the barriers scale), the three questionnaire scales displayed adequate psychometric properties with ordered response thresholds, no misfitting items or DIF, and importantly, minimal evidence of multidimensionality. There was also slight mistargeting of item difficulty to person ability, especially for the confidence questionnaire, indicating that on average, participants found the questions slightly too “difficult”. Further iterative deletion of items in all three questionnaire scales unfortunately resulted in worse fit statistics and we therefore decided to stop and export these person measures for use in subsequent parametric testing.

*Supplementary Table 1. Psychometric properties of the three scales utilised in the study*

|  | Rasch model | Action in Practice | Revised Action in Practice | Confidence | Revised Confidence | Barriers | Revised Barriers |
| --- | --- | --- | --- | --- | --- | --- | --- |
| Items |  | 1-7 | 1-2, 4-7 | 1-11 | 1-4, 6-8, 10 | 1-13 | 1-2,4-8, 10-11 |
| Disordered thresholds | No | No | No | No | No | No | No |
| Person separation index | >2.0 | **1.69** | **1.71** | 2.66 | 2.55 | **2.01** | **1.79** |
| Person reliability | >0.8 | **0.74** | **0.75** | 0.88 | 0.87 | **0.80** | **0.76** |
| PCA, variance by 1^st^ factor | >50% | 62.1% | 66.7% | 56% | 66.1% | **48.1** | **44.2** |
| PCA, Eigenvalue for 1^st^ contrast | <2.0 | 1.58 | 1.64 | **2.61** | **2.07** | **2.46** | 2.0 |
| Item fit (infit MnSq) | <1.3 | None | None | **Item 11** | None | **Item 9** | None |
| Differential item functioning (DIF) contrast* | <1.0 logits and P<0.05 | **Item 3 (DIF for previous depression training)** | None | **Item 5 (DIF for previous depression training)** | None | **Items 3, 9, 12 (DIF for previous depression training)** | None |
| Targeting, difference between person & item means | <1.0 logits | **-1.60** | **-1.84** | **-1.63** | **-1.87** | -0.37 | **-0.85** |
| *DIF was assessed for age group, gender and previous depression training  Bolded values indicate suboptimal fit to the Rasch model | | | | | | | |

**References**

1. Nguyen TH HH, Kim MT, Chan KS. An introduction to item response theory for patient-reported outcome measurement. Patient. 2014;7(1):23-35.
