# Supplementary material for: How do community-based eye care practitioners approach depression in patients with low vision? A mixed methods study
Source: BMC Psychiatry. 2019 Dec 30;19:426. doi: 10.1186/s12888-019-2387-x (PMC6937690; doi:10.1186/s12888-019-2387-x)
Supplement: Supplementary file 3 — Additional file 3. Responses to action in practice scale. Figure S1. indicates the responses to all action in practice scale items. [file 12888_2019_2387_MOESM3_ESM.docx]

**Additional File 3 – Supplementary Figure 1.**

Supplementary Figure 1. indicates the actions taken in response to suspected depression.


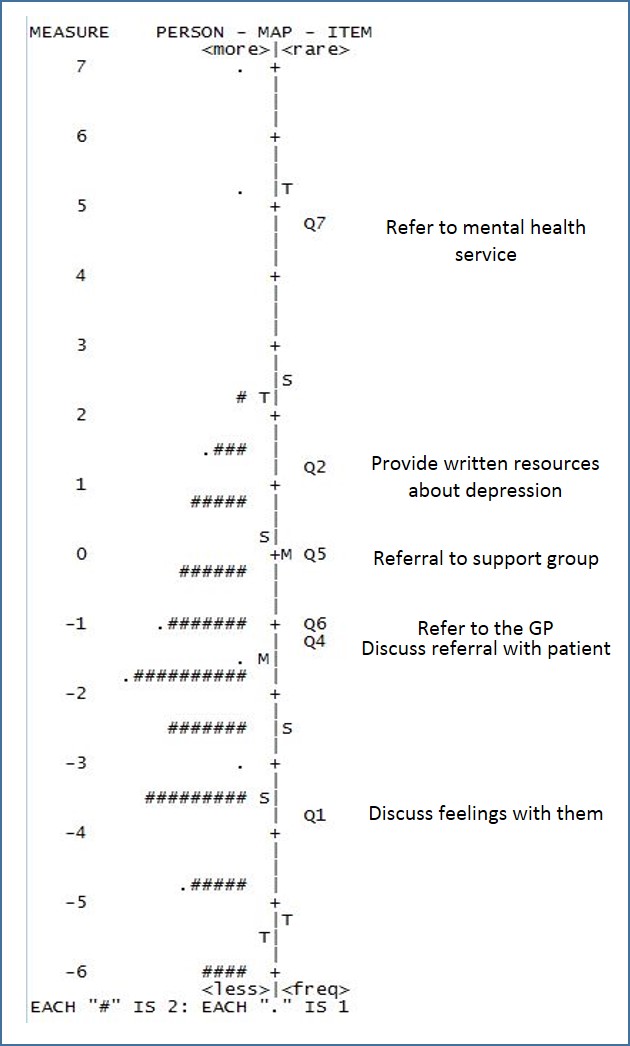


*Practitioners are represented on the left of the dashed line, with “#” equivalent to 2 people and “.” equivalent to 1 person. The items are represented on the right of the dashed line. The items at the top represent actions that practitioners took rarely, while the items at the bottom are actions practitioners took more frequently.*
