# Supplementary material for: How do community-based eye care practitioners approach depression in patients with low vision? A mixed methods study
Source: BMC Psychiatry. 2019 Dec 30;19:426. doi: 10.1186/s12888-019-2387-x (PMC6937690; doi:10.1186/s12888-019-2387-x)
Supplement: Supplementary file 4 — Additional file 4. Responses to confidence scale. Figure S2. indicates responses to all confidence scale items. [file 12888_2019_2387_MOESM4_ESM.docx]

**Additional File 4 – Supplementary Figure 2.**

Supplementary Figure 2. indicates practitioners’ confidence in working with patients with low vision


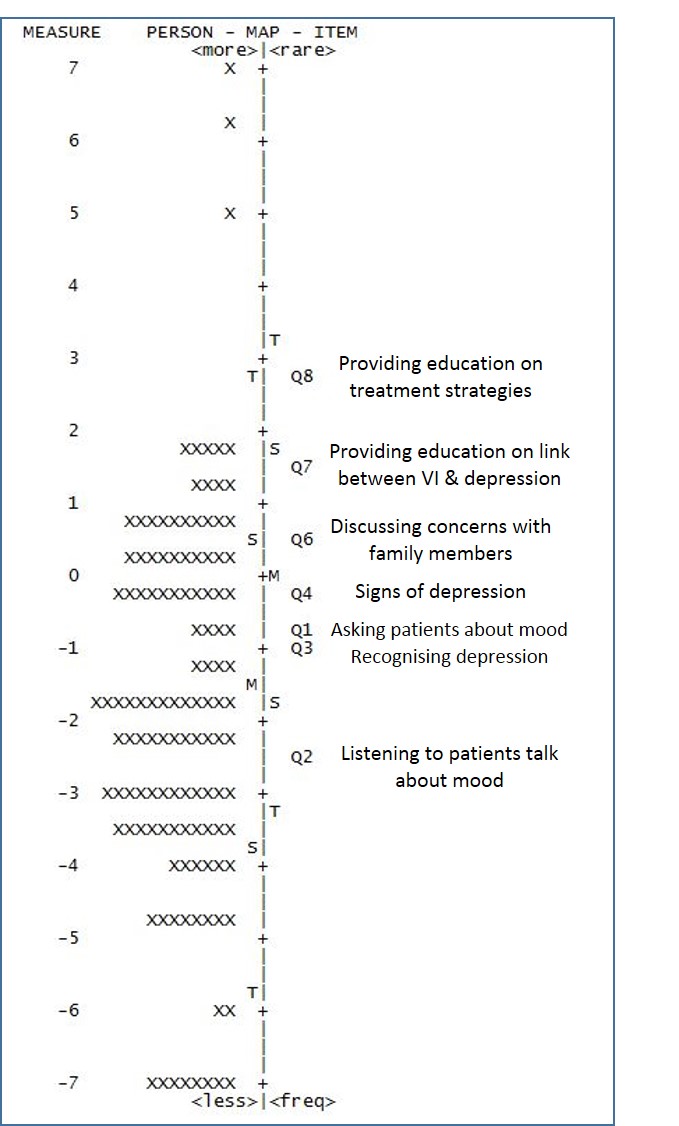


*Practitioners are represented on the left of the dashed line, with “x” equivalent to 1 person. Items are represented on the right of the dashed line - those at the top are ones that practitioners were least confident about and the items at the bottom of the map were those practitioners felt more confident about.*
