# Supplementary material for: How do community-based eye care practitioners approach depression in patients with low vision? A mixed methods study
Source: BMC Psychiatry. 2019 Dec 30;19:426. doi: 10.1186/s12888-019-2387-x (PMC6937690; doi:10.1186/s12888-019-2387-x)
Supplement: Supplementary file 5 — Additional file 5. Responses to barriers scale. Figure S3. indicates the responses to all barrier scale items. [file 12888_2019_2387_MOESM5_ESM.docx]

**Additional File 5 – Supplementary Figure 3.**

Supplementary Figure 3. indicates the perceived barriers to working with patients with low vision.


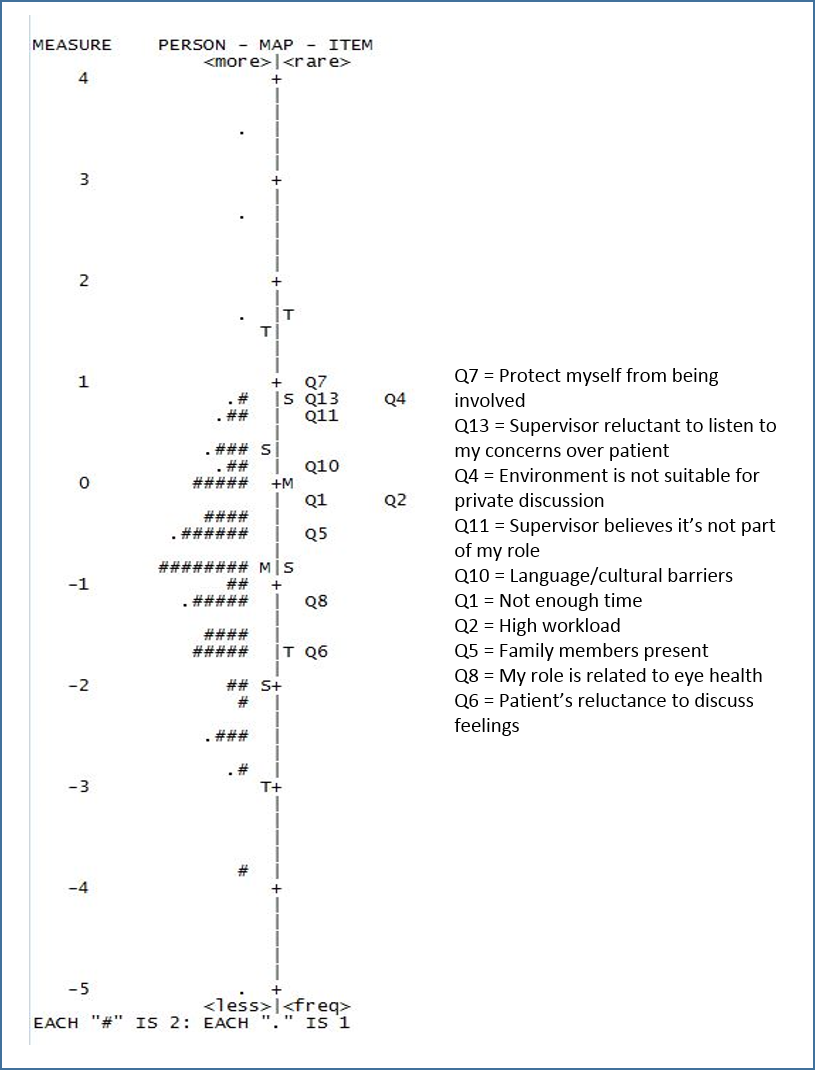


*Practitioners are represented on the left of the dashed line, with “#” equivalent to 2 people and “.” equivalent to 1 person. Items are represented on the right of the dashed line with items at the top representing the barriers that practitioners reported least frequently, while items at the bottom of the map were barriers that practitioners reported most frequently.*
